# Perceptual suppression of predicted natural images

**DOI:** 10.1167/16.13.6

**Published:** 2016-10-31

**Authors:** Rachel N. Denison, Jacob Sheynin, Michael A. Silver

**Affiliations:** rachel.denison@nyu.edu www.racheldenison.com; jacob.sheynin@mail.mcgill.ca; masilver@berkeley.edu http://argentum.ucbso.berkeley.edu; Helen Wills Neuroscience Institute, University of California, Berkeley, Berkeley, CA, US,; Department of Psychology and Center for Neural Science, New York University, New York, NY USA; College of Letters and Science, University of California, Berkeley, Berkeley, CA, USA; Helen Wills Neuroscience Institute, Vision Science Graduate Group, and School of Optometry, University of California, Berkeley, Berkeley, CA, USA

**Keywords:** *prediction*, *expectation*, *perceptual selection*, *binocular rivalry*, *visual statistical learning*

## Abstract

Perception is shaped not only by current sensory inputs but also by expectations generated from past sensory experience. Humans viewing ambiguous stimuli in a stable visual environment are generally more likely to see the perceptual interpretation that matches their expectations, but it is less clear how expectations affect perception when the environment is changing predictably. We used statistical learning to teach observers arbitrary sequences of natural images and employed binocular rivalry to measure perceptual selection as a function of predictive context. In contrast to previous demonstrations of preferential selection of predicted images for conscious awareness, we found that recently acquired sequence predictions biased perceptual selection toward unexpected natural images and image categories. These perceptual biases were not associated with explicit recall of the learned image sequences. Our results show that exposure to arbitrary sequential structure in the environment impacts subsequent visual perceptual selection and awareness. Specifically, for natural image sequences, the visual system prioritizes what is surprising, or statistically informative, over what is expected, or statistically likely.

## Introduction

How do our previous experiences influence what we see? Many studies have demonstrated that predictions based on past experience can affect sensory processing and perception (reviewed in Panichello, Cheung, & Bar, 2013). Visual history can influence not only the speed and efficiency of processing, it can also change *what* we see. Prior experience can bias our perception of visual features and objects (Chalk, Seitz, & Seriès, 2010; Fischer & Whitney, 2014; Gibson & Radner, 1937; Liberman, Fischer, & Whitney, 2014). Moreover, when visual input is ambiguous, prior experience can influence perceptual selection: that is, which perceptual interpretation is represented in conscious awareness and which interpretation is suppressed and remains unseen (Brascamp, Knapen, Kanai, van Ee, & van den Berg, 2007; Chopin & Mamassian, 2012; Denison, Piazza, & Silver, 2011; Haijiang, Saunders, Stone, & Backus, 2006; Long, Toppino, & Mondin, 1992; Maloney, Dal Martello, Sahm, & Spillmann, 2005; Pearson & Brascamp, 2008; Sterzer, Frith, & Petrovic, 2008; Wolfe, 1984). Here, to better understand the dynamic processes that determine the contents of consciousness, we investigated the effects of prediction on visual perceptual selection.

According to Bayesian theories, perception reflects a combination of current perceptual input and prior expectations (Kersten, Mamassian, & Yuille, 2004; Snyder, Schwiedrzik, Vitela, & Melloni, 2015), such that perceptual selection should favor predicted interpretations (Hohwy, Roepstorff, & Friston, 2008; Seriés & Seitz, 2013). For example, one general prediction is that the future visual environment will be similar or identical to the current one (Weiss, Simoncelli, & Adelson, 2002; Wiskott & Sejnowski, 2002). Consistent with the Bayesian framework in this case, many studies have found that perceptual selection favors both repeated percepts (Leopold, Wilke, Maier, & Logothetis, 2002; Pearson & Brascamp, 2008; Pearson, Clifford, & Tong, 2008) and percepts associated with repeated context (Di Luca, Ernst, & Backus, 2010; Haijiang et al., 2006; Schmack et al., 2013; Sterzer et al., 2008). Perceptual selection also favors perceptual interpretations predicted by preceding stimulus motion (Attarha & Moore, 2015; Denison et al., 2011; Maloney et al., 2005).

An important exception to the prediction hypothesis is adaptation, which reduces the likelihood of maintaining one's current percept (Alais, Cass, O'Shea, & Blake, 2010; Blake & Overton, 1979; Chopin & Mamassian, 2012). Indeed, repeated percepts are especially favored when adaptation is not a major factor. This occurs, for example, when the preceding stimulus is brief or weak, such that adaptation is minimal (Brascamp et al., 2007; Pearson et al., 2008).

To date, all of the studies that reported prediction effects on perceptual selection have used relatively simple stimuli that were made of dots or gratings. However, much of the predictability in our visual input in natural environments is based on complex visual sequences, such as a planned sequence of saccades, walking along a familiar route, or watching someone perform a series of actions. Such complex sequences may recruit different predictive mechanisms than the simpler sequences and stimuli that have been previously studied. To investigate the effects of a broader range of dynamic predictions on perceptual selection, we employed visual statistical learning of natural images and binocular rivalry. This method does not require a limited stimulus set and is not restricted to predictions based on repetition or motion.

Statistical learning is the rapid acquisition of knowledge about patterns in the sensory environment (Fiser & Aslin, 2001). It has been demonstrated for arbitrary image sequences, even when observers are not aware of any patterns (Brady & Oliva, 2008; Fiser & Aslin, 2002; Turk-Browne, Jungé, & Scholl, 2005; Turk-Browne & Scholl, 2009). Through statistical learning, observers in our study acquired novel predictive sequences of natural images. We then asked how the predictions generated by these sequences influenced subsequent perceptual selection during binocular rivalry. Binocular rivalry (Wheatstone, 1838) is a sensitive probe of perceptual decision processes and a powerful tool for studying perceptual selection (Alais & Blake, 2005; Blake & Logothetis, 2002). During rivalry, different images are presented to the two eyes, and perception alternates between the two images in a way that is largely outside of the observer's control (Meng & Tong, 2004).

We further examined whether any effects of statistical learning on perception would be tied to a specific level of visual processing (Brady & Oliva, 2008) (individual images vs. image categories) and whether the level involved would depend on which types of image features were attended (Turk-Browne et al., 2005). To investigate these questions, we tested perceptual selection for both image and category sequences and directed participants' attention towards either images or categories during their initial exposure to the sequences.

We found that perception was indeed influenced by the predictions associated with the learned sequences. However, in contrast to the Bayesian framework, observers were more likely to perceive the unexpected image and category. That is, predicted images/categories were perceptually suppressed by unexpected images/categories. These findings indicate that the visual system can prioritize unexpected items in complex visual sequences. Our sequence prediction protocol provides a way to determine how the visual system balances two competing strategies: incorporating prior information (favoring predicted percepts) versus selecting the most statistically surprising visual information (favoring nonpredicted percepts).

## Methods

### Observers

Sixty-one naïve observers participated in the experiments. There were 18 observers in each of three experimental groups (E1, image exposure task: 18–25 years; four male,14 female; E2, category exposure task: 18–31 years; five male,13 female; E3, category exposure task with short exposure phase: 18–33 years; five male,13 female). These sample sizes are comparable to those of previous studies on statistical learning (Fiser & Aslin, 2001) and on the influence of predictive information on binocular rivalry (Denison et al., 2011). Two additional observers were tested in E2, but their data could not be used due to a technical error that prevented responses from being recorded. Five additional observers participated in the equivalent contrast experiment (19–31 years, one male, four female). All observers provided informed consent, and all experimental protocols were approved by the Committee for the Protection of Human Subjects at the University of California, Berkeley, and were conducted in accord with the Declaration of Helsinki.

### Visual stimuli

Stimuli were generated on a Macintosh PowerPC using Matlab and Psychophysics Toolbox (Brainard, 1997; Pelli, 1997) and were displayed on two halves of a gamma-corrected NEC MultiSync FE992 CRT monitor with a refresh rate of 60 Hz at a viewing distance of 100 cm. Observers viewed all stimuli through a mirror stereoscope with their heads stabilized by a chin rest. Visual stimuli were natural images presented within circular patches 1.8° in diameter and were surrounded by a black annulus with a diameter of 2.6° and a thickness of 0.2°. Binocular presentation of this annulus allowed it to serve as a vergence cue to stabilize eye position and to ensure that the rivaling stimuli were presented to corresponding retinal locations in the two eyes. All stimuli were presented on a neutral gray background (luminance of 59 cd/m^2^). Images were grayscale photographs that were histogram equalized so that all images had an identical distribution of pixel luminances and the same mean luminance as the background. During rivalry presentations, the image presented to one eye was tinted red and the image presented to the other eye was tinted blue by increasing the values in either the red or the blue channel by 50%.

**Figure 1:**
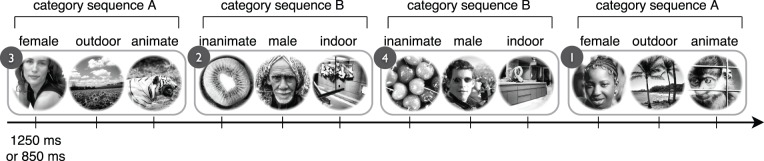
Image sequence exposure to induce statistical learning. Observers viewed an image stream composed of 12 unique images grouped into four triplet sequences (triplet numbers shown in circles), presented in pseudorandom order. Each triplet sequence was one of two types of category sequence (A or B). Observers received no cues to indicate the triplet structure of the streams. Part of an example stream including all four triplets is shown.

Twelve natural images were selected to form four three-image sequences (triplets), with two images coming from each of six categories (male, female, animate, inanimate, indoor scene, outdoor scene). The entire image set is shown in Figure 1. The third images from sequences 1 and 2 and the third images from sequences 3 and 4 were paired during the rivalry test phase. Since not all images rival equally with one another, the paired images were chosen by screening a large set of images to find two animate/indoor scene pairs in which the rivaling images had approximately equal levels of initial dominance in binocular rivalry. The images in the first and second positions of each triplet, which were never used as rivalry stimuli, were selected more arbitrarily.

Photographs were obtained from a commercial digital library (Corel Stock Photo Libraries from Corel Corporation), the Berkeley Segmentation Dataset (http://www.eecs.berkeley.edu/Research/Projects/CS/vision/grouping/segbench/) and, with permission, from the personal collection of Kendrick Kay.

### Overview of procedure

Each experimental session had four parts: (a) an exposure phase, to induce statistical learning of triplet image sequences; (b) a rivalry test, to determine the impact of sequence learning on perceptual selection in binocular rivalry; (c) a recall test, to determine to what extent observers noticed and could explicitly recall the triplets shown during the exposure phase; and (d) a familiarity test, to determine to what extent observers could recognize the triplets in a forced-choice setting.

### Exposure phase

#### Stimuli

A stream of grayscale natural images was presented identically to the two eyes through a mirror stereoscope. Images were presented one at a time, in sequence, and were organized into triplets that had both image- and category-level structure (Figure 1). The presentation order of the triplets was pseudorandomized, with the constraint that no triplet could be presented twice in a row within the sequence. This constraint reduced the likelihood that observers would notice the triplet structure within the image stream. There were no cues to indicate the stream's triplet structure.

#### Task

Observers performed either an image identification task (E1) or a category identification task (E2/E3), directing their attention during the exposure phase to either lower-level image or higher-level category information, respectively. They were instructed to press a key corresponding to the image identity (E1) or category identity (E2/E3) of each presented image as quickly and accurately as possible.

#### Procedure

Observers first completed short practice blocks to learn the response key mappings until they either achieved 85% identification accuracy or completed five blocks. Each practice block consisted of six presentations of each triplet with a slow image presentation rate (2000 ms/image in E1 and 1250 ms/image in E2/E3, 0 ms interstimulus interval [ISI]).

Next, observers completed exposure blocks that contained 18 presentations of each triplet (E1 and E2: five exposure blocks, or 90 presentations of each triplet; E3: two exposure blocks, or 36 presentations of each triplet). Each image was presented for 1250 ms (E1) or 850 ms (E2/E3) with 0 ms ISI. The rate of image presentation was slower in E1 than in E2 and E3 because the image identification task (12 possible responses) was more difficult than the category identification task (six possible responses).

### Rivalry test

#### Design

**Figure 2:**
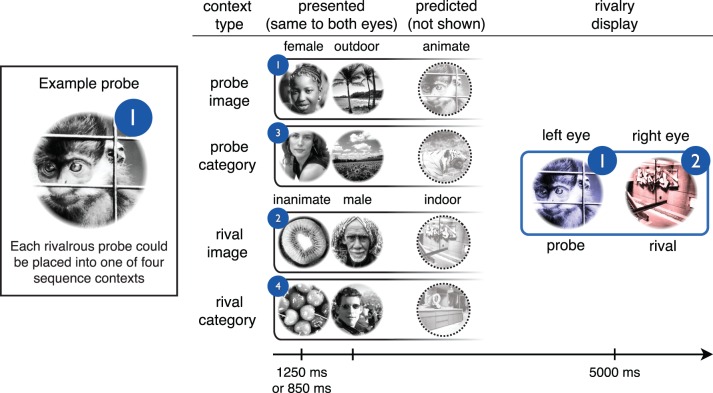
Binocular rivalry test. The third image from each triplet was used as a probe during the binocular rivalry test. Each rivalry display was preceded by the first two images from one of the triplets, creating four types of sequence context. In *probe image* and *probe category* trials, the first two images in the sequence predicted either the same image or the same category as the probe, respectively. In *rival image* and *rival category* trials, the first two images in the sequence predicted either the same image or category as the probe's rival, respectively. This design enabled perceptual selection of a given image in a given rivalrous pair to be measured as a function of predictive sequence context. Circled numbers show the triplet containing each image or sequence (c.f. Figure 1).

On each trial of the rivalry test (Figure 2), two images from one of the four triplets (the “sequence context”) were followed by a rivalry display in which the image in one eye was predicted by the first two images, whereas the image in the other eye was not. To maintain experimental control over stimulus factors that can influence binocular rivalry, we manipulated sequence context while keeping the rivalry displays consistent across trials. The rivalry display was always one of two rivalrous pairs: the third images from triplets 1 and 2, or the third images from triplets 3 and 4 (see Figure 1 for triplet sequences).

We label experimental conditions according to the relationship between the sequence context and a given rivalrous image, or “probe,” with condition names indicating what was predicted by the sequence context. Thus, in the “probe image” condition, the sequence context predicted the appearance of the probe image (i.e., the probe came from the same triplet as the context). In the “rival image” condition, the sequence context predicted the appearance of the image rivaling with the probe (the “rival”). In the “probe category” condition, the sequence context predicted neither the probe nor the rival image, but rather a third image (from the other rivalrous pair) that shared the same category as the probe. In the “rival category” condition, the sequence context predicted a different image that shared the same category as the rival.

If statistical learning promoted perception of predicted images and/or categories, there would be greater rivalry dominance of probes during the “probe image” and/or “probe category” conditions. Conversely, if statistical learning promoted perception of unexpected images and/or categories, there would be greater dominance of probes during the “rival image” and/or “rival category” conditions, in which rivals were predicted by the sequence context, and probes were unexpected.

#### Stimuli

On each trial, the two sequence context images were presented binocularly in grayscale, followed by a rivalry display (Figure 2). The rivalry images were tinted red and blue (one color in each eye), which reduced piecemeal rivalry percepts and allowed observers to report the perceptually dominant image based on its color. Tint colors and left and right eye presentations for the images in each of the two rivalry pairs were counterbalanced across trials.

#### Task

Observers passively viewed the grayscale images and then reported the dominant percept during rivalry by holding down one of two keys, corresponding to the red and blue tints, for as long as the corresponding percept persisted. They were instructed not to press any key for ambiguous percepts.

#### Procedure

On each trial, the two sequence context images were presented binocularly with the same timing as in the exposure phase (1250 ms in E1 or 850 ms in E2/E3, 0 ms ISI). The rivalry display was then presented for 5 s. This duration was sufficient for the initiation of unambiguous rivalry and typically at least one perceptual alternation, enabling measurement of first response duration.

Observers completed two runs of the rivalry task. Each run consisted of 96 trials presented in blocks of 32 trials. Each of the four rivalrous images appeared in 48 trials during the run, 12 times in each sequence context condition. Thus, there were 48 data points per condition per run, with all trials randomly intermixed.

#### Analysis

We expected that predictive sequence context effects would be strongest at the beginning of the rivalry period of each trial, so our primary analysis was of the initial response to the rivalry stimuli—the proportion of trials in which the initial percept was the probe versus the rival. We also measured the latency and duration of the initial response for both probe and rival percepts. The relatively short rivalry presentation durations we employed did not allow analysis of responses following the initial percept (see Denison et al., 2011 for a similar approach).

### Recall test

#### Procedure

Observers were interviewed to assess their recall of image and category sequence regularities during the exposure phase. They were first asked to describe any patterns they had noticed in the image streams during the exposure phase. Following this open-ended questioning, they were specifically prompted to report any repeated temporal sequences they might have noticed, both for specific images and for categories. The following specific prompts were given: “For example, what image generally followed the man with the sunglasses?” (image sequence prompt) and “For example, what kind of image generally followed a man?” (category sequence prompt). Observers were encouraged to report as many sequences as they could remember.

#### Analysis

We calculated the recall rate for each observer (number of sequences recalled divided by total number of sequences presented during the exposure phase), separately for image and category sequences. Very few complete triplet sequences were recalled by observers (see Results), so to increase sensitivity for detecting a correlation between recall and the effects of statistical learning on rivalry responses, we included both triplets and sequential pairs of images and categories from the exposure stream in the recall measure.

For each observer, the recall rates for triplets (out of 4) and pairs (out of 8) were calculated separately and then added together to give the total recall rates for image and category sequences. Triplet and pair counts were mutually exclusive in order to avoid double-counting (i.e., recall of a triplet was not also counted as recall of two pairs). So, for example, if an observer identified 4/4 triplets (and thus 0/8 pairs), the score would be 1. If an observer identified 3/4 triplets and 1/8 pairs (the maximum number of pairs that could be recalled, since the final triplet was not correctly identified), the score would be 0.875. If an observer identified 0/4 triplets, the maximum possible score would be 4/8 pairs, or 0.5. Thus, the recall rate could range from 0 (no recall) to 1 (perfect recall of all four triplets). The overall recall rate for each observer was defined as the average of the image and category recall rates.

### Familiarity test

#### Design

Following the interview, observers were informed that, indeed, some sequences of images had appeared more often than others during the exposure phase. Observers then performed a familiarity task (Figure 4a, b) that tested their ability to discriminate three types of sequences: triplets from the exposure phase, *category-match* foils, and *category-different* foils.

**Figure 3:**
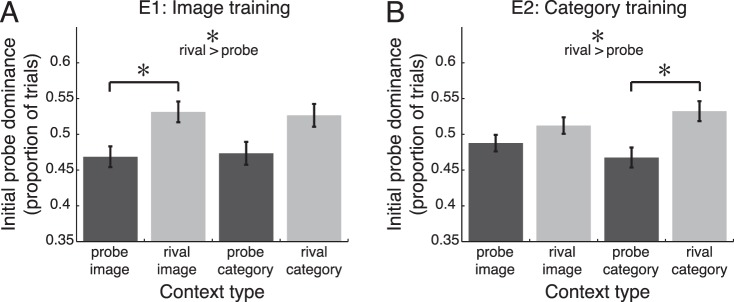
Previously established sequence context enhances perceptual selection of unexpected compared to predicted images and categories. Dark bars represent trials in which sequence context predicted the probe image or category, and light bars represent trials in which context predicted the rival image or category. (A) Rivalry responses when an image identification task was performed during exposure. (B) Rivalry responses when a category identification task was performed during exposure. Error bars are *SEM* across observers; *n* = 18 observers in each experiment. *, *p* < 0.05.

**Figure 4:**
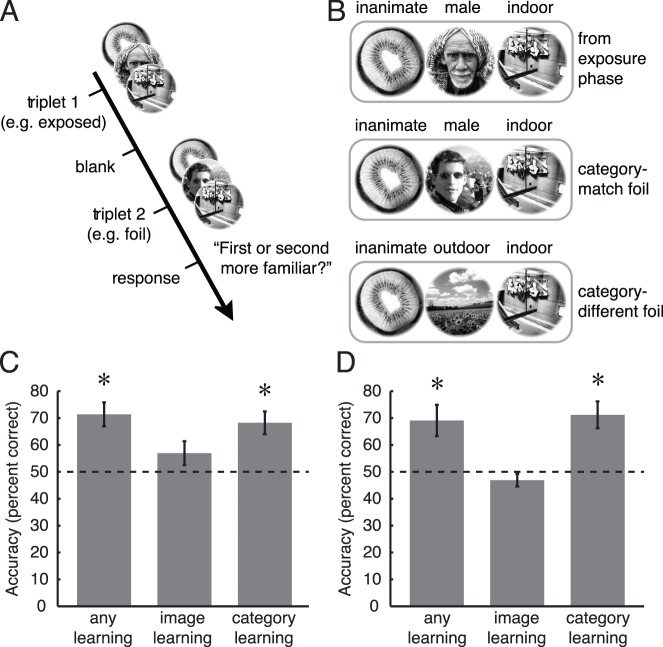
Familiarity task results. (A) On each trial, observers reported which of two three-image sequences was more familiar (2AFC) based on presentations during the exposure phase. (B) Learning of image sequence, category sequence, and any type of sequence (image or category) was assessed by employing category-match foils and category-different foils that had not previously been shown to observers. (C) 2AFC accuracy for the three types of learning in E1. Chance performance was 50%. (D) 2AFC accuracy in E2. Error bars are *SEM* across observers; *n* = 18 observers in each experiment. *, *p* < 0.005.

There were four category-match and four category-different foil sequences, none of which had been seen by the observers during either the exposure or rivalry test phases. Foil sequences were identical to the triplets that were presented during the exposure phase, except that the second image of each foil was replaced by an image from a different triplet. For category-match foils, the replacement image came from the same category as the original image. For category-different foils, the replacement image came from a different category than the original image.

Employing these two types of foils allowed us to assess three kinds of learning, as measured by recognition performance. *Image learning* was measured when one sequence was from the exposure phase and the other was a category-match foil. If observers had only learned category information and not specific image information, they should have performed poorly in discriminating these two sequences. *Category learning* was measured when one sequence was a category-match foil and the other was a category-different foil. If observers had learned category information, they should have been able to discriminate these foils, but if they had only learned image information, they would have been unable to do so, since neither of the foil sequences had been shown during the exposure phase. Finally, in *any learning* trials, one sequence was an original triplet from the exposure phase, and the other was a category-different foil. Either image or category learning would have enabled observers to discriminate these two sequences.

#### Stimuli

Stimuli were grayscale image sequences, identical to those used in the exposure phase.

#### Task

Two triplets were presented sequentially on each trial, and observers reported whether the first or the second sequence was more familiar (2AFC).

#### Procedure

The timing of each sequence presentation was the same as in the exposure phase. The two sequences were separated by a 1-s blank period. A 3-s response window followed the second sequence presentation. For each of the three conditions (image learning, category learning, and any learning), each of the four triplets of one sequence type was paired with each triplet of the other sequence type two times (counterbalanced order of triplet presentation), resulting in a block of 96 randomly intermixed trials (32 trials for each condition).

#### Analysis

We measured the proportion of correct 2AFC responses in each condition, where the correct response was defined as the option that had previously appeared as an image sequence, a category sequence, or both (i.e., the original triplet in the any learning and image learning conditions and the category-match foil in the category learning condition).

### Estimation of equivalent contrast

In a separate experiment, we estimated the size of the predictive rivalry effect in units of image contrast. The goal was to determine how much the contrast of a rivaling image would have to be reduced to have the same proportion of initial perceptual selection as when it was predicted by one of the learned sequences.

#### Stimuli and task

We varied the Michelson contrast of one eye's image (variable contrast image; contrasts = 0.2, 0.5, 0.7, 0.8, 0.85, 0.9, 0.95, 1) while keeping the contrast of the other eye's image fixed at 1 (standard image). The contrast response function was densely sampled at high contrasts because contrasts generating proportions of initial responses that were equivalent to the magnitude of the predictive rivalry effect were expected to be in that range. The rivalrous image pairs were the same as those used in the rivalry test.

#### Procedure

All aspects of the equivalent contrast procedure were the same as in the rivalry test, except only the rivalry display was presented (i.e., it was not preceded by sequence context). The standard and variable contrast image assignments were counterbalanced across images, red/blue tints, and eyes. Each observer completed 1024 trials (128 per contrast), with all trial types randomly intermixed, in two, one-hour sessions.

#### Analysis

For each observer, the proportion of trials for which the variable contrast image was selected as the initial percept in rivalry was plotted as a function of contrast on a logarithmic scale, and a cumulative normal function was then fit to this contrast response function. The mean of the function was fixed to be zero—log(1)—and the value of the psychometric function at that contrast was fixed to be 0.5, because there should be no overall preference for the variable versus the standard contrast images when both have a contrast of 1. The standard deviation (width) and the amplitude of the function were free parameters. This function fit the data well: R^2^ for individual observers ranged from 0.90–0.98 (mean = 0.96). For each observer, the fitted function was used to estimate the image contrast corresponding to the size of the prediction effect measured in the rivalry test. This was the contrast at which the value of the fitted function equaled the mean proportion of trials in which predicted images were initially selected. The mean and standard error of this equivalent contrast were then calculated across the five observers.

## Results

### Exposure phase

Experimental groups E1 and E2 completed the same experiment under different task conditions during the exposure phase. In E1, observers identified specific images, and in E2, they identified categories. Exposure consisted of 90 repetitions of each triplet.

Image and category identification accuracy during the exposure phase was well above chance but not at ceiling, indicating that the tasks were sufficiently difficult to engage attention. Accuracy was similar for the two experiments [mean percent correct across observers, E1: 77% (*SD* = 14%; chance performance = 8%); and E2: 77% (*SD* = 12%; chance performance = 17%)].

### Binocular rivalry

Given the possibility that perceptual biases due to statistical learning would change over the course of the rivalry test, we included experimental run as a factor in ANOVAs for each experiment. The effect of statistical learning on initial perceptual dominance during binocular rivalry was different for the first and second run for E1 [repeated-measures, three-way ANOVA with factors of run, probe/rival context, and image/category context; interaction between run and probe/rival context: *F*(1, 17) = 7.71, *p* = 0.013, 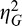 = 0.094] and marginally so for E2, [*F*(1, 17) = 3.03, *p* = 0.100, 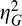 = 0.049]. We therefore analyzed the two runs separately. There were no other significant effects in the full experiment ANOVAs (all *F* < 2.5, *p* > 0.13).

In the first run of the rivalry test (Figure 2), perceptual selection during binocular rivalry was influenced by prior statistical learning of the image and category sequences. Regardless of whether observers performed the image or category identification task during exposure, we observed a main effect of probe versus rival context, such that observers were more likely to perceive a given probe when its rival image or category was predicted by the first two images shown in the trial [Figure 3; repeated-measures, two-way ANOVA with factors of probe/rival context and image/category context, E1: *F*(1, 17) = 7.20, *p* = 0.016, 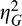 = 0.18; E2: *F*(1, 17) = 4.55, *p* = 0.048, 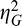 = 0.15]. In other words, the predicted item was more likely to be initially *suppressed* during rivalry, while the unexpected item was more likely to be perceptually dominant.

The average difference in proportion initial dominance for predicted and nonpredicted images and categories across the two experiments was 0.051. That is, the predicted image was selected 47.4% of the time. We performed an additional experiment in which we determined the reduction in image contrast that would decrease the proportion of initial rivalry responses to that level (lowering contrast decreases dominance in binocular rivalry [Levelt, 1965]). We found that the average predictive rivalry effect shown in Figure 3 is equivalent to a 5.5% reduction in image contrast (*SEM* = 0.78%; see Methods, Estimation of equivalent contrast).

We did not find significant probe versus rival context effects in the second run of the rivalry task [repeated-measures, two-way ANOVA with factors of probe/rival context and image/category context, E1: *F*(1, 17) = 0.83, *p* = 0.37, 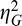 = 0.027; and E2: *F*(1, 17) = 0.21, *p* = 0.66, 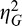 = 0.006]. It is possible that the effects of statistical learning from the exposure phase degraded due to the lack of consistent triplet presentation over the two rivalry test runs. All subsequent analyses of rivalry data report results from the first run.

We next directly compared *probe image* versus *rival image* and *probe category* versus *rival category* conditions in the two experiments. In E1 we found a significant effect of statistical learning on perceptual selection at the image level, with greater initial dominance of the unexpected image [paired *t* test, probe versus rival image, *t*(17) = 2.17, *p* = 0.045, *d* = 0.51], but no reliable difference between probe and rival category conditions [*t*(17) = 1.67, *p* = 0.11, *d* = 0.28] (Figure 3). Conversely, in E2 we found a significant rivalry effect at the category level, with more initial dominance of the unexpected category [paired *t* test, probe versus rival category, *t*(17) = 2.33, *p* = 0.033, *d* = 0.55] and no reliable effect at the image level [*t*(17) = 1.06, *p* = 0.30, *d* = 0.25] (Figure 3). However, there were no significant interactions between probe/rival and image/category factors in either experiment [repeated-measures, two-way ANOVA, E1: *F*(1, 17) = 0.051, *p* = 0.82, 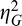 = 0.0015; and E2: *F*(1, 17) = 1.88, *p* = 0.19, 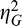 = 0.035].

Combining the two experiments (mixed three-way ANOVA with experiment as a between-subjects factor) again showed more initial dominance for unexpected images and categories [main effect of probe/rival, *F*(1, 34) = 11.64, *p* = 0.0017, 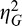 = 0.16] but no differential rivalry effect for images and categories in the two experiments [no three-way interaction between probe/rival, image/category, and experiment, *F*(1, 34) = 0.92, *p* = 0.34, 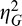 = 0.011].

Because we expected predictive effects to be strongest at the beginning of each presentation of rivaling stimuli, our primary measure of interest was the initial percept, which reflects the outcome of the first perceptual selection between the competing alternatives. We also measured the mean duration and latency of the initial response, which index rivalry processes that are distinct from those that determine the identity of the initial percept, and found that these were not influenced by statistical learning. In particular, there were no significant effects of image sequence context on initial response duration [repeated-measures, two-way ANOVA, E1: main effects and interaction *F*(1, 17) < 1.50, all *p* > 0.2; and E2: main effects and interaction *F*(1, 17) < 1.67, all *p* > 0.2] or latency [repeated-measures, two-way ANOVA, E1: main effects and interaction *F*(1, 17) < 1.70, all *p* > 0.4; and E2: main effects and interaction *F*(1, 17) < 2.66, all *p* > 0.1]. We obtained the same results for initial response duration when we excluded from the analysis those initial key presses that continued until the end of the 5-s rivalry duration. This dissociation between predictive context effects on the identity and the duration of the initial percept in binocular rivalry is consistent with previous work indicating different processes underlying perceptual selection and maintenance in rivalry (Bressler, Denison, & Silver, 2013; de Jong, Knapen, & van Ee, 2012; Levelt, 1965; Sobel & Blake, 2002; Stanley, Forte, Cavanagh, & Carter, 2011).

### Familiarity and recall

In addition to determining the effects of statistical learning on perceptual selection in rivalry, we separately assessed statistical learning using familiarity and recall tasks that measured both image and category learning (see Methods). In the familiarity task (Figure 4), we found evidence for strong category learning but little image learning. The any learning measure, reflecting a combination of image and category learning, was well above chance (50%) performance in both experiments [one-sample *t* test, E1: *t*(17) = 4.78, *p* < 0.001, *d* = 1.13; and E2: *t*(17) = 3.27, *p* = 0.0045, *d* = 0.77]. The same was true for category learning in both experiments [E1: *t*(17) = 4.33, *p* < 0.001, *d* = 1.02; and E2: *t*(17) = 4.23, *p* < 0.001, *d* = 1.00], even though in E1, observers were never provided with category labels and never performed category judgments during the exposure phase.

Mean accuracy in the category learning condition was similar to mean accuracy in the any learning condition, suggesting that most of the learning measured by the familiarity task was at the category level in both experiments. Indeed, we did not find reliable evidence for image learning in either experiment [E1*: t*(17) = 1.55, *p* = 0.14, *d* = 0.37; E2: *t*(17) = 1.34, *p* = 0.20, *d* = 0.32]. Moreover, familiarity judgments for image sequences were less accurate than those for category sequences in both experiments [paired *t* test, E1: *t*(17) = 2.65, *p* = 0.017, *d* = 0.62; E2: *t*(17) = 5.19, *p* < 0.001, *d* = 1.22]. Greater familiarity for category compared to image sequences may have been at least partially due to the hierarchical relationship between images and categories, as each category contained two distinct images, resulting in twice as many presentations of each category sequence as of each image sequence.

**Figure 5:**
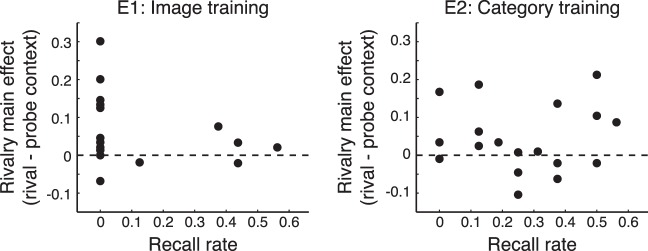
Recall results and correlation with statistical learning effects on rivalry. Relationship between the magnitude of the rivalry main effect (the difference between probe and rival context, averaged across image/category) and sequence recall (obtained from interviews) across observers in E1 (left) and E2 (right). Data points represent individual observers. Recall rate would be 1 for perfect recall and 0 for no recall of any image and/or category sequences (see Methods).

Despite clear evidence of statistical learning as assessed with the familiarity task, there were low rates of triplet recall. Even with prompting (see Methods), no observer correctly recalled a complete image triplet sequence in either experiment. On average, recollection of complete category triplets was 3% in E1 and 28% in E2. There was some additional recall of partial image and category sequences (i.e., sequential image pairs representing part of a triplet), and we quantified this by computing a recall rate for each observer in which a value of 0 indicates that no sequence information was recalled, and a value of 1 represents complete recall (Methods and Figure 5). Mean recall rates were 0.13 (*SD* = 0.21) for E1 and 0.27 (*SD* = 0.18) for E2.

We found no positive correlation between recall rate and the main effect of sequence context on perceptual selection in binocular rivalry (Figure 5) [E1: *r*(16) = −0.32, *p* = 0.20, CI = [-0.68 0.17]; E2: *r*(16) = 0.04, *p* = 0.88, CI = [-0.44 0.50]]. Given that strong eye dominance can reduce the effects of experimental manipulations on the outcome of rivalry we recomputed these correlations while controlling for a measure of eye dominance in each observer (defined as the proportion of trials that the initial percept was the image in the dominant eye). Again, there was no significant correlation between recall rate and the magnitude of the rivalry effect [E1: *r*(16) = −0.26, *p* = 0.31; E2: *r*(16) = −0.0003, *p* = 0.99].

Indeed, we found no correlations for any pairwise combination of our three measures of statistical learning effects: rivalry, familiarity, and recall. Correlations between measures across individuals were not significant for the rivalry main effect of probe/rival context versus the any learning familiarity measure [E1: *r*(16) = 0.11, *p* = 0.66, CI = [-0.37 0.55]; E2: *r*(16) = 0.067, *p* = 0.79, CI = [-0.41 0.52]] or for any learning versus recall rate [E1: *r*(16) = 0.42, *p* = 0.080, CI = [-0.054 0.74]; E2: *r*(16) = 0.19, *p* = 0.44, CI = [-0.30 0.61]]. These results are generally consistent with previous work that also found no correlation of different learning measures across observers following visual statistical learning (Kim, Seitz, Feenstra, & Shams, 2009).

### Short exposure

One factor that may influence whether selection favors the predicted versus unexpected image or category is the amount of previous exposure to the sequences. For example, infants look longer at more familiar stimuli early in an exposure period but longer at novel stimuli after more prolonged exposure (Rose, Gottfried, Melloy-Carminar, & Bridger, 1982). Therefore, we tested whether a shorter exposure phase (36 repeats per triplet) would reverse the direction of the rivalry effect, resulting in increased perceptual selection of predicted images and/or categories (E3; categorization task during exposure).

**Figure 6:**
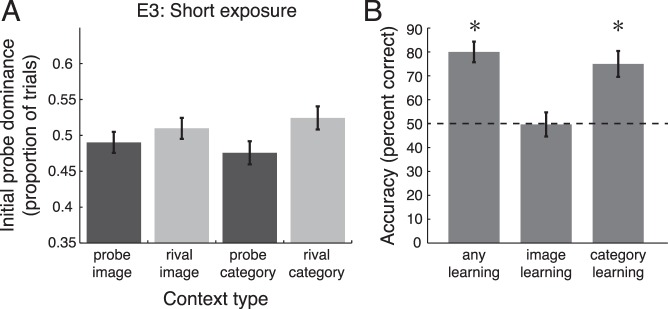
Short exposure experiment (E3). (A) Initial probe dominance as a function of sequence context (conventions as in Figure 3). (B) Familiarity test performance (conventions as in Figure 4). Error bars are *SEM* across observers; *n* = 18 observers. *, *p* < 0.001.

Mean accuracy on the exposure task was 69% (*SD* = 19%, chance = 17%). In the rivalry test, the difference between probe and rival context was in the same direction as in E1 and E2, but it had a lower magnitude and did not reach significance: Figure 6A; repeated-measures, two-way ANOVA with factors of probe/rival context and image/category context, *F*(1, 17) = 2.08, *p* = 0.17, 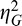 = 0.067. These results are not consistent with shorter exposures leading to perceptual selection of predicted images or categories. The reduced effect size in E3 compared to E1 and E2 is expected if the effects of statistical learning on subsequent rivalry accrue over the exposure period. The familiarity test confirmed that the shorter exposure duration in E3 was sufficient for statistical learning as it is typically measured in a 2AFC recognition task [Figure 6B; any learning: *t*(17) = 6.93, *p* < 0.001, *d* = 1.63.] As in E1 and E2, we found category learning, *t*(17) = 4.63, *p* < 0.001, *d* = 1.09, but no image learning, *t*(17) = −0.069, *p* = 0.95, *d* = 0.016. Mean recall rate was 0.20 (*SD* = 0.18).

## Discussion

We found enhanced perceptual selection of images that violated the predictable sequential structure contained in previously exposed image streams. This is contrary to the notion that “we see what we expect to see,” an idea that has received experimental support from several previous studies of perception of ambiguous visual displays. Rivalry repetition (Leopold et al., 2002; Pearson & Brascamp, 2008), visual imagery (Pearson et al., 2008), learned cue associations (Haijiang et al., 2006; Sterzer et al., 2008), predictable sequences of dot motion (Maloney et al., 2005), and predictive rotational motion (Attarha & Moore, 2015; Denison et al., 2011) have all been shown to bias perception of ambiguous displays in a direction consistent with expectations derived from the accompanying context. These findings support the view that recent visual history can serve as a prior that combines with incoming sensory information to determine the contents of perception (Bressler et al., 2013; Denison et al., 2011; Kersten et al., 2004).

However, only seeing what we expect to see neglects events that prompt us to update our knowledge of the visual world. From a statistical perspective, surprising events contain more information about the environment than predicted ones. Therefore, the brain may balance Bayesian inference with prioritization of new information. Enhancing processing of new information in the environment is often offered as a functional explanation for adaptation and its perceptual consequences (McDermott, Malkoc, Mulligan, & Webster, 2010; Ranganath & Rainer, 2003). Our results show that the visual system can also exhibit biases that enhance perception of violations of sequences.

The bias toward unexpected stimuli in perceptual selection and conscious awareness that we report is conceptually related to studies of statistical learning and attentional capture that show enhanced processing of surprising stimuli. In statistical learning, cortical responses to image sequences are enhanced for surprising compared to predicted images. Physiological studies of macaque inferotemporal cortex found that exposure to image sequences resulted in reduced neuronal responses to images in the later part of the sequence, compared to unexpected images (Meyer & Olson, 2011; Meyer, Ramachandran, & Olson, 2014). Cortical responses in inferotemporal cortex, a high-level, object-selective region, also correlate strongly with perception during binocular rivalry in the macaque (Sheinberg & Logothetis, 1997). Therefore, reduced responses to predicted images in higher order visual cortex may be tied to perceptual suppression of those images during rivalry. The involvement of higher-level visual areas would be consistent with our finding of effects of statistical learning on rivalry at the category level.

Unexpected images have also been found to capture attention (Brockmole & Henderson, 2005; Foley, Jangraw, Peck, & Gottlieb, 2014; Näätänen, 1990). Statistical learning can influence the allocation of attention (Cosman & Vecera, 2014; Zhao, Al-Aidroos, & Turk-Browne, 2013), and exogenous feature-based attention can impact binocular rivalry (Chong & Blake, 2006), especially at its onset (Mitchell, Stoner, & Reynolds, 2004). Increased attention to a nonpredicted stimulus may therefore increase perceptual selection of that stimulus. In rivalry studies, natural scenes containing atypical, as opposed to typical, objects are selected more quickly (Mudrik, Breska, Lamy, & Deouell, 2011) and have extended periods of perceptual dominance (Mudrik, Deouell, & Lamy, 2011), findings which the authors suggested were due to increased attention to the unexpected images.

Combining statistical learning and attention, Zhao et al. (2013) found faster reaction times for visual search targets embedded in streams of triplet sequences that had been statistically learned compared to targets embedded in streams of random sequences at other spatial locations. The authors concluded that attention was drawn to the location with learned sequential structure. An alternative interpretation of these results, however, is that attention was drawn toward sequence violations. That is, targets may have attracted attention because they were not part of the original learned sequences and therefore violated the sequential structure of the sequences during search. The two interpretations of this study are not mutually exclusive, as attention can be allocated in space, as Zhao et al. suggest, as well as in time, triggered by a sequence violation.

Taken together, these statistical learning and attention studies in which unexpected images were prioritized used natural images (or images of complex objects), nonmotion sequences, or both, as in our study. Therefore, one or both of these factors may be key to our observation of surprise effects rather than the prediction effects observed in most previous studies of the influence of prediction on perceptual selection. Note that motion may be a special case for the visual system, which is highly specialized for motion processing (Berry, Brivanlou, Jordan, & Meister, 1999), and predictive extrapolations of motion have been observed in a variety of perceptual and behavioral phenomena (Freyd & Finke, 1984; Nijhawan, 1994; Roach, McGraw, & Johnston, 2011).

Further studies will be required to test whether the use of natural images and/or nonmotion sequences are critical for the preferential selection of surprising percepts and to examine the relationships among this surprise effect, associated brain responses, and attention. Additional manipulation of experimental parameters such as image content, stimulus timing, and exposure duration will help to determine the generality of our findings. E3 provides some initial constraints on the required exposure duration. In addition, E1 and E2 suggest that once established, the rivalry effects dissipate over time, possibly due to exposure to new sequences during the rivalry test that are inconsistent with those presented during the exposure phase. Overwriting of statistical learning could reflect ongoing tracking of stimulus statistics by the visual system.

Our study is also related to theoretical accounts of the effects of prediction on perception. In predictive coding models (Friston, 2005; Rao & Ballard, 1999), higher order cortical areas generate predictions regarding the activity of lower order areas. Studies motivated by predictive coding have often focused on top-down predictions generated from spatial (or otherwise concurrent) context (Alink, Schwiedrzik, Kohler, Singer, & Muckli, 2010; Rao & Ballard, 1999; Smith & Muckli, 2010) or from stable expectations (Egner, Monti, & Summerfield, 2010; Summerfield et al., 2006), but a dynamical implementation of predictive coding has been used to explain surprise effects such as the mismatch negativity (Wacongne, Changeux, & Dehaene, 2012). For rivalrous perception following a predictive sequence of the kind used here, the behavior of dynamical predictive coding models would depend both on the implementation of these longer timescales and on the level(s) in the visual hierarchy that were associated with perception during rivalry (Hohwy et al., 2008).

Recently, the concept of a perceptual “continuity field” has been proposed, based on findings of perceptual biases toward previously presented stimuli (Fischer & Whitney, 2014). These attractive biases last for several seconds and are somewhat location-specific. Such “serial dependence” has been observed for orientation (Fischer & Whitney, 2014), numerosity (Corbett, Fischer, & Whitney, 2011), and face (Liberman et al., 2014) perception, thus spanning multiple levels of stimulus complexity. Continuity fields in perception are reminiscent of rivalry memory (Leopold et al., 2002), a stabilizing bias in perceptual selection, as both are evident at delays at which adaptation has largely dissipated (Chopin & Mamassian, 2012). The surprise effects we have observed in rivalry are opposite to those predicted by a continuity field, and resolution of this discrepancy is an interesting direction for future research.

## Conclusions

We found that violations of predictable natural image sequences were preferentially selected for conscious awareness. There are two main implications of this finding. First, perceptual selection can be shaped by arbitrary sequential patterns in the environment. Our work extends the range of known predictive effects on awareness to include patterns that are commonly found in natural environments. Second, predictions can facilitate perception of unexpected stimuli in the absence of low-level adaptation. Thus, in determining the contents of conscious experience, the human perceptual system appears to use two complementary strategies: a Bayesian integration of the past and present and a surprise-based selection of unexpected information. Our flexible sequence prediction protocol establishes an experimental approach for investigating how the visual system balances these two strategies to optimize perception in dynamic environments.

## Supplementary Material


