# Supplementary figures and images for: Perceptual suppression of predicted natural images
Source: J Vis. 2016 Oct 31;16(13):6. doi: 10.1167/16.13.6 (PMC5098454; doi:10.1167/16.13.6)

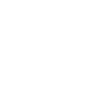

Supplement: Supplementary file 1 [file JOV-05269-2016-s01-ICON.gif]
